# Comparison of Tonsillectomy Complication Rates in a Global Underserved Community to Those of a Tertiary Medical Center

**DOI:** 10.1055/s-0046-1817803

**Published:** 2026-03-17

**Authors:** Russell A. Whitehead, Evan A. Patel, Julio A. Roque Buenrostro, Regina Elmudesi del Rio, Isamar Fernandez, Bryan A. Himmel, Stephanie Crane, Ashok Jagasia

**Affiliations:** 1 Department of Otolaryngology – Head and Neck Surgery, Rush University Medical Center, Chicago, IL, United States; 2 School of Medicine, Universidad Iberoamericana, Santo Domingo, Dominican Republic; 3 Department of Internal Medicine, Rush University Medical Center, Chicago, IL, United States

**Keywords:** tonsillectomy, adenoidectomy, postoperative complications, surgical trip, global health

## Abstract

**Introduction:**

Understanding surgical outcomes in diverse settings is crucial for safe delivery of healthcare to underserved communities. Insufficient research has been done analyzing postoperative complications in regions with limited access to healthcare resources.

**Objective:**

To compare postoperative complications from tonsillectomies and adenoidectomies on global health trips to an underserved community to those of a tertiary academic medical center (TAMC).

**Methods:**

Data was collected via phone survey with patients from 2 2023 surgical trips to Azua, in the Dominican Republic (DR), sponsored by a TAMC, including surgery type, postoperative healthcare visits, and postoperative complications. The same data was collected via retrospective chart review from patients at a TAMC in 2023. The surgeries included were tonsillectomy adenoidectomy (T&A), tonsillectomy (T), and adenoidectomy (A). A statistical analysis compared non-routine healthcare visits and postoperative complications between populations using the Fisher's exact test.

**Results:**

In the DR, 168 patients (mean age: 8 years) underwent T&A (n = 113), T (n = 34), and A (n = 21). At the TAMC, 520 patients (mean age: 7.8 years) underwent T&A (n = 288), T (n = 123), and A (n = 109). No patients from the DR reported postoperative bleeding within 2 weeks of surgery, compared to 23 patients from the TAMC ( *p* < 0.001). There was no statistical difference in trouble swallowing and uncontrolled pain within 2 weeks between the populations ( *p* = 0.673 and 0.465).

**Conclusion:**

The T&A procedures on global health trips to underserved communities were not associated with increased rates of life-threatening complications compared with a TAMC. Surgical trips can provide safe and effective otolaryngologic care to patients in underserved communities.

## Introduction

Tertiary academic medical centers (TAMCs) and international aid organizations commonly sponsor surgical trips to underserved areas of the world with scarce resources. 1 Thousands of operations are preformed yearly by such organizations and include subspecialities such as orthopedics, urology, ophthalmology, and otolaryngology. 2 3 Surgical trips are commonly mission-driven, with the goal of addressing healthcare disparities and improving access to surgical interventions in regions with limited resources and expertise. 4 5 While these trips can have a positive impact by providing essential medical care to those in need, they also raise ethical considerations, including patient safety in the postoperative period. 6 As a result, it is essential to approach such endeavors with cultural sensitivity, respect for local customs, and a commitment to long-term collaboration and support.

The Dominican Republic (DR) is a low-income country in the Caribbean with a population of approximately 10.8 million people and poor access to healthcare, specifically otolaryngology. 7 8 In the capital city, Santo Domingo, there are approximately 65 otolaryngologists serving a population of 3.5 million, and this specialized care is not included in the nation's healthcare coverage. 9 As a result, otolaryngologic care is extremely limited in the DR, especially in regions outside the capital city, necessitating frequent surgical trips by TAMCs and international organizations to provide basic care to patients, including tonsillectomy (T), adenoidectomy (A), tonsillectomy adenoidectomy (T&A), pressure equalizing (PE) tubes, and facial plastic reconstructive surgery.

Understanding surgical outcomes in diverse settings is crucial for safe delivery of healthcare to underserved communities. However, minimal research has been done analyzing patient safety and postoperative complications on surgical trips due to limited opportunity for follow-up and limited access to local healthcare. 10

Thus, the goal of the present study is to compare the rates of postoperative complications from T&As on surgical trips in the DR to those of a TAMC in order to have a more holistic understanding of underserved communities' safety on such trips.

## Methods

Azua, an underserved city in the southern region of the DR, is the site of a biannual otolaryngology surgical trip sponsored by a TAMC. Prior to each surgical trip, the senior author (AJ) worked closely with a charitable organization stationed in the DR, Community Empowerment, to recruit new patients to the local hospital. Partnering with local bilingual medical personnel ensured that Spanish interpretation was not a barrier for screening patients and obtaining assent/consent. Verbal consent was obtained from parents/guardians prior to patient screening as the current study involved pediatric patients. Institutional Review Board (IRB) exemption was granted by the TAMC's review board, and consent was obtained from Community Empowerment on behalf of the local DR hospital.

Retrospective data was collected postoperatively via phone surveys after 2 surgical trips to the DR in 2023, sponsored by a TAMC. All patients from the DR included in the study underwent T&A, tonsillectomy (T), or adenoidectomy (A) and were operated on by TAMC otolaryngologists working out of a local hospital on two separate week-long surgical trips. The DR cohort included both adult and pediatric patients. Surgical technique involved the use of cautery in the DR and cautery/coblation at the TAMC.

Data collected on postoperative phone surveys included patient age, preoperative (preop) diagnosis (i.e., recurrent tonsillitis, hypertrophic adenoids/tonsils, and sleep disordered breathing, among others), preoperative symptoms (i.e., breathing problems, snoring, recurrent upper respiratory infections, recurrent ear infections, and trouble swallowing, among others), operation performed (i.e., T&A, T, or A), and non-routine postoperative healthcare visits at 2-week and 3-month intervals.

Non-routine postoperative visits were further divided by reasons related to surgery (i.e., uncontrolled bleeding, trouble swallowing, uncontrolled pain) and unrelated. Identical data was collected via retrospective chart review at a TAMC on patients who underwent identical operations in 2023. The Current Procedural Terminology (CPT) codes were queried on both adult and pediatric patients at the TAMC who had undergone T&A, T, and A for similar indications (recurrent tonsillitis, sleep disordered breathing, malignancy). Statistical analysis compared postoperative complications and non-routine healthcare visits between populations using Fisher's exact test. A *p* -value < 0.05 was considered significant throughout statistical analysis.

## Results

In the DR, 168 patients with a mean age of 8 years underwent otolaryngologic surgery on 2 surgical trips in 2023. There were 113 patients who underwent T&A, 34 underwent T, and 21 underwent A. Comparing it to the TAMC, 520 patients were chart-reviewed who underwent identical operations in 2023, with a mean age of 7.8 years. There were 288 patients who underwent T&A, 123 underwent T, and 109 underwent A in 2023.

For the DR, frequently reported preoperative symptoms included trouble swallowing (n = 95), swollen throat (n = 94), and heavy snoring (n = 78). The most common preoperative diagnoses were recurrent tonsilitis (n = 66), hypertrophic tonsils, and adenoids (n = 87), and sleep disordered breathing (n = 23). For the TAMC, heavy snoring was the most common presenting symptom (n = 303), followed by recurrent infections (n = 153), and difficulty breathing (n = 90). The most common preoperative diagnoses were sleep-disordered breathing (n = 248), hypertrophic tonsils (n = 218), and obstructive sleep apnea (n = 129). A summary of all demographic data including preop symptoms and diagnoses can be found in Table 1 .

**Table 1:** Demographics of all patients who underwent otolaryngologic surgery

Patients	DR	TAMC
**Demographics**		
Total	168	520
Pediatric	141	407
Adults	27	113
Mean age	8	7.8
Median age	7	7
**Operation**		
Tonsillectomy and adenoidectomy	113	288
Tonsillectomy	34	123
Adenoidectomy	21	109
**Preoperative symptoms**		
Heavy snoring	78	303
Recurrent infections	68	153
Difficulty breathing	68	90
Recurrent ear infections	3	62
Swollen throat	94	47
Trouble swallowing	95	41
**Preoperative diagnosis**		
Sleep disordered breathing	23	248
Hypertrophic tonsils	12	218
Hypertrophic tonsils and adenoids	87*	129
Obstructive sleep apnea	-**	129
Hypertrophic adenoids	21*	117
Recurrent tonsilitis	66	107
Cancer/Suspected cancer	0	6

**Abbreviations:** DR, Dominican Republic; TAMC, tertiary academic medical center. **Notes:** *All patients diagnosed with hypertrophic adenoids in the DR presented with lateral neck x-ray at initial consultation. **No sleep studies were completed in the DR to allow for diagnosis of OSA.

Postoperatively, 2 DR patients reported seeking non-routine medical care within 2 weeks of surgery, compared with 55 from the TAMC ( *p* < 0.001). By age, 1 pediatric DR patient and 38 pediatric TAMC patients sought non-routine care within 2 weeks of surgery ( *p* < 0.001). There was 1 adult DR patient and 17 from the TAMC who sought non-routine care within 2 weeks ( *p* = 0.197). At 2-week postoperatively, visits were then further divided by complaints related to surgery and unrelated to surgery. There were 2 DR patients who sought non-routine care within 2 weeks for reasons related to surgery, compared with 34 from the TAMC ( *p* = 0.005). By age, 1 pediatric DR patient and 17 from the TAMC sought non-routine care within 2 weeks for reasons related to surgery ( *p* = 0.054). Furthermore, 1 adult DR patient and 17 from the TAMC sought non-routine care within 2 weeks for reasons related to surgery ( *p* = 0.197). A summary of non-routine 2-week postoperative visits can be found in Table 2 .

**Table 2:** Summary of all non-routine visits within 2-weeks of surgery

Patients	DR	TAMC	*p* -value
**All non-routine 2-week postoperative visits**			
Total	2	55	**< 0.001**
Pediatric patients	1	38	**< 0.001**
Adult patients	1	17	0.197
**Non-routine 2-week postoperative visits related to surgery**			
Total	2	34	**0.005**
Pediatric patients	1	17	0.054
Adult patients	1	17	0.197

**Abbreviations:** DR, Dominican Republic; TAMC, tertiary academic medical center.

No DR patients reported postoperative bleeding, but 23 TAMC ones experienced it ( *p* = 0.002). Of these 23 patients, 13 (56.5%) were taken back to the operating room to control the bleeding, 5 (21.7%) had their bleeding managed in clinic or the emergency room and were discharged home, and 5 (21.7%) required overnight admission to the hospital but were not managed surgically. Furthermore, 13 (56.5%) had a diagnosis of recurrent tonsillitis. There were 2 DR patients who reported trouble swallowing compared to 4 from the TAMC ( *p* = 0.6733). Also, 1 DR patient reported uncontrolled pain, compared to 9 TAMC patients ( *p* = 0.465). All DR patients presented to a local clinic for evaluation and did not require emergency department care, hospital admission, or revision surgery. A summary of non-routine 2-week postoperative visits for complaints related to surgery can be found in Table 3 .

**Table 3:** Summary of all nonroutine visits within 2-weeks of surgery for complaints related to surgery

Patients	DR	TAMC	*p* -value
**2-week postoperative visits related to hemorrhage**			
Total	0	23*	**0.002**
Pediatric	0	9	0.121
Adult	0	14	0.072
**2-week postoperative visits for other complaints related to surgery**			
Trouble swallowing	2	4	0.638
Uncontrolled pain	1	9	0.465

**Abbreviations:** DR, Dominican Republic; Post-op, postoperative; TAMC, tertiary academic medical center.

**Note:** *13 patients required operating room to resolve bleeding.

No patients in the DR reported seeking non-routine medical care within 2 weeks of surgery for reasons other than a surgical complication, while 21 TAMC patients did. Common complaints included otalgia (n = 6), upper respiratory tract infection (URI, n = 3), fever (n = 3), vomiting (n = 2), dehydration (n = 2), cough (n = 2), epistaxis (n = 1), shortness of breath (n = 1), and rash (n = 1). All of these patients were managed at local clinics and emergency departments, and none of them required hospital readmission. At the 3-month postoperative interval, no patients from the DR (n = 168) or the TAMC (n = 520) reported seeking non-routine medical care for reasons related to their surgery.

## Discussion

The present study included patients from Azua, an underserved community in Southern DR with extremely limited access to otolaryngologic care and instruments, including bipolar cautery, and radiofrequency coblation, as well as limited access to a pediatric intensive care unit. The local population relies on routine surgical trips sponsored by TAMCs to receive the necessary otolaryngologic care.

In 2023, 168 patients from the DR underwent tonsillectomy and/or adenoidectomy on surgical trips compared to 520 patients from a TAMC, with a very similar mean age (8 and 7.8 years, respectively) and similar preoperative diagnoses, including hypertrophic tonsils/adenoids and sleep disordered breathing. Post-tonsillectomy hemorrhage is a relatively common but potentially life-threatening complication, known to occur in up to 5% of patients. 11

Those who underwent operations in the DR did not experience increased rates of life-threatening complications (postoperative bleeding) from T&As when compared to TAMC patients. In fact, patients at the TAMC experienced higher rates of postoperative bleeding, likely due to higher surgical volume. Additionally, DR patients reported rates of non-life-threatening postoperative complications that were not significantly different from the ones at the TAMC, including difficulty swallowing, and uncontrolled pain. Our data indicates that operations provided on surgical trips do not put patients at increased risks of postoperative complications, ensuring that proper sterile and safety protocols are adhered to.

Surgical trips to underserved areas of the globe increase access to healthcare and provide essential services to patients who would not otherwise be able to obtain affordable care. 2 8 12 It was found that patients from a TAMC sought non-routine postoperative healthcare at higher rates in the short-term (2-weeks postoperatively) for reasons related and unrelated to surgery. This is likely due to their increased access to local otolaryngologic and primary care. Additionally, there was no difference in non-routine postoperative healthcare visits related to surgery in the long-term (3-months postop) between populations.

Common long-term complications from tonsillectomy may include problems with throat secretions, throat clearing, dysphagia, and problems with voice or speech. 13 The results of our study reassure that patients in both settings equally do not seek care for these problems in the long-term.

Few previous studies have examined postoperative complication rates on surgical trips to underserved areas of the globe. The existing literature describes how plastic surgery trips are not associated with increased complication rates, 14 and there is limited patient follow-up after orthopedic surgical trips. 15 16 It has been demonstrated that patients who undergo head and neck operations on surgical trips report effective procedures and improved quality of life. 3 17 The existing literature also highlights the importance of involving local providers to strengthen infrastructure, promote education, and provide culturally competent care. 18 19 However, to the best of our knowledge, our study is the first to compare complications of T&As on surgical trips to underserved communities. Our data suggests that such operations are just as safe as when performed at a TAMC, with no increased rates of life-threatening postoperative complications.

Our study has numerous limitations that should be addressed. While this is the first study to examine T&A postoperative complication rates in an underserved community compared with a TAMC, collecting data via phone survey presents a magnitude of challenges. Phone surveys in low-resource areas with limited follow-up options are highly prone to underreported complications. Without access to medical documentation, patients' answers, as well as those by parents/guardians, were subject to recall bias, which may have influenced the reported outcomes. Future studies should aim to mitigate this bias, such as engaging local healthcare providers in data collection to improve accuracy. Additionally, not all patients who underwent operations answered the phone survey, even after numerous attempts at contact, subjecting this data to selection bias.

While collecting data via phone survey certainly presents vast challenges, it represents the most feasible option given the constraints and limited resources in the area. Furthermore, preoperative diagnoses of DR patients were somewhat limited due to lack of lateral neck x-rays and lack of proper sleep studies. It is also important to address the fact that although our data demonstrated a lower rate of complications in an underserved community compared to a TAMC, which is likely related to a lack of access to healthcare facilities to report any complications that may have occurred. While this lack of access is why these communities are selected for medical mission trips to begin with, it may limit our findings. On the contrary, TAMC patients likely have easier access to care, potentially inflating reported complications. Despite such limitations, we believe our data provides an encompassing overview of rates of postoperative complications in both patient populations.

Surgical trips play vital roles in addressing limited access to medical care in low-income countries. Additionally, they contribute to medical education, as they frequently involve training local healthcare professionals and fostering collaboration between medical teams and local providers. 20 Driven by a humanitarian commitment to improve quality of life, surgical trips promote global health equity, social responsibility, and empower local communities. 21

## Conclusion

Tonsillectomy and adenoidectomy on global health trips to an underserved community in the DR were not associated with increased rates of life-threatening complication when compared with performing these procedures at a TAMC. Patients from the TAMC sought non-routine postoperative healthcare at higher rates in the short-term, likely due to easier access. Surgical trips can be safe and effective at providing otolaryngologic care to patients in underserved communities.

## References

[1] Lin B M Varvares M A Randolph G W Shaye D A United States-based global otolaryngology surgery: A call to more horizontal sustainable efforts Am J Otolaryngol 2019 40 03 404 408 10.1016/j.amjoto.2019.02.012 30799209

[2] McQueen K A Hyder J A Taira B R Semer N Burkle F M Jr Casey K M The provision of surgical care by international organizations in developing countries: a preliminary report World J Surg 2010 34 03 397 402 10.1007/s00268-009-0181-5 19685261

[3] Panuganti B A Jafari A Shen S Predictors of Quality-of-Life Improvements Following Global Head and Neck Surgery Trips to Underserved Regions Laryngoscope 2021 131 09 2006 2010 10.1002/lary.29522 33734447

[4] Tollefson T T Shaye D Durbin-Johnson B Mehdezadeh O Mahomva L Chidzonga M Cleft lip-cleft palate in Zimbabwe: estimating the distribution of the surgical burden of disease using geographic information systems Laryngoscope 2015 125 01 S1 S14 10.1002/lary.24747 24867649

[5] Chweya C M Ryder C Y Fei-Zhang D J Bidirectional needs assessment of otolaryngology-head and neck surgery short-term surgical trips to Ethiopia and Kenya Laryngoscope Investig Otolaryngol 2023 8 01 303 312 10.1002/lio2.1014 PMC9948567 36846413

[6] Luginbuhl A Kahue C N Stewart M Head and neck surgery global outreach: Ethics, planning, and impact Head Neck 2021 43 06 1780 1787 10.1002/hed.26643 33586258 PMC8248027

[7] Bureau U SC World Population Clock: Dominican Republic Washington Census Bureau 2024. Available at:https://www.census.gov/popclock/world/dr

[8] Urban M J Wojcik C Losenegger T Incorporating hearing screening to an otolaryngology surgical mission in the rural Dominican Republic Int J Pediatr Otorhinolaryngol 2022 160 111222 10.1016/j.ijporl.2022.111222 35839652

[9] Goulios H Patuzzi R B Audiology education and practice from an international perspective Int J Audiol 2008 47 10 647 664 10.1080/14992020802203322 18923986

[10] Wiedermann J Douse D M Green K J Outcomes of Short-Term Surgical Trips in Otolaryngology-Head and Neck Surgery: A Scoping Review Laryngoscope 2024 134 01 32 39 10.1002/lary.30764 37249184

[11] Wall J J Tay K Y Postoperative Tonsillectomy Hemorrhage Emerg Med Clin North Am 2018 36 02 415 426 10.1016/j.emc.2017.12.009 29622331

[12] Prasad K Peterson N Nolen D Building a sustainable free flap program in a resource-limited setting: A 12-year humanitarian effort Head Neck 2024 46 05 1051 1055 10.1002/hed.27640 38233973

[13] Odhagen E Alm F Axelsson S Long-term complications after tonsil surgery: an analysis of 54,462 patients from the Swedish Quality Register for Tonsil Surgery Front Surg 2023 10 1.304471E6 10.3389/fsurg.2023.1304471 PMC10749945 38148748

[14] Sue G R Deptula P L Chang J Surgical Team Trips to Vietnam: Implementation of a Dedicated Cleft Palate Perioperative Program Improves Fistula Rates Ann Plast Surg 2021 87 05 528 532 10.1097/SAP.0000000000002795 33661215

[15] Leversedge C Castro S Appiani L MC Kamal R Shapiro L Patient Follow-up After Orthopaedic Outreach Trips - Do We Know Whether Patients are Improving? World J Surg 2022 46 10 2299 2309 10.1007/s00268-022-06630-w 35764890 PMC9436850

[16] Torchia M T Schroder L K Hill B W Cole P A A Patient Follow-up Program for Short-Term Surgical Mission Trips to a Developing Country J Bone Joint Surg Am 2016 98 03 226 232 10.2106/JBJS.O.00087 26842413

[17] Richard K Sanchez R Amado B Pediatric Otolaryngology Short-Term Mission Outcomes at a Surgical Mission Hospital in Guatemala Otolaryngol Head Neck Surg 2024 170 01 252 259 10.1002/ohn.432 37466003

[18] Al-Hadidi A Alslaim H Ghawanmeh M Short-term surgical trips: local collaboration and its effects on complications and patient satisfaction Pediatr Surg Int 2020 36 08 977 981 10.1007/s00383-020-04667-3 32415355

[19] Billig J I Nasser J S Chung W HJ Huynh K A Chung K C Education during Surgical Outreach Trips in Vietnam: A Qualitative Study of Surgeon Learners Plast Reconstr Surg Glob Open 2020 8 07 e2969 10.1097/GOX.0000000000002969 32802662 PMC7413802

[20] Jafari A Tringale K R Campbell B H Husseman J W Cordes S R Impact of Humanitarian Experiences on Otolaryngology Trainees: A Follow-up Study of Travel Grant Recipients Otolaryngol Head Neck Surg 2017 156 06 1084 1087 10.1177/0194599817691274 28301300

[21] Rule A RL Warrick S Rule D W Using Reflective Writing to Explore Resident Resilience during Global Health Electives Am J Trop Med Hyg 2022 106 03 923 929 10.4269/ajtmh.21-0682 35008047 PMC8922490

